# Effects of Scapular Kinetic-Chain Exercise on Muscle Activity in Overhead-Pitching Baseball Players

**Published:** 2020-05

**Authors:** Ki-Jae SONG, Jin-Ho YOON, Jae-Keun OH

**Affiliations:** 1. Department of Sports Medicine, Korea National Sport University, Seoul, Korea; 2. Department of Sports Rehabilitation, Korea Nazarene University, Cheonan, Korea

**Keywords:** Kinetic-chain, Muscle activity, Scapular dyskinesis, Scapular kinetic-chain exercise

## Abstract

**Background:**

We aimed to demonstrate the effect of 8 wk of scapular kinetic-chain exercise on muscle activity in collegiate baseball players diagnosed with scapular dyskinesis.

**Methods:**

The subjects were career baseball players with at least a 7-year career assigned into either a SICK (S; scapular malposition, I; inferior border prominence, C; coracoid process pain, K; scapular dyskinesis; n=7) group or a normal group (n=16), respectively. The groups were further divided into SICK-Dominant, SICK-Non-Dominant, Normal-Dominant, and Normal-Non-Dominant to examine the dominant and non-dominant deviation of each group. Twenty-three subjects finally recruited through the Sports Science Institute of Korea National Sport University, Seoul, Korea in Sep 2014. Subjects were only allowed drug treatment for acute injuries during matches or training, and matches, training, and diet were controlled by university dormitory life.

**Results:**

There was a significant increase in maximal muscular activation (MA) in elevation motion of Normal-Dominant upper trapezii (UT), mean MA of SICK-Dominant UT, and mean MA of SICK-Dominant lower trapezii (LT) (*P*<0.05). In depression motion, the mean MAs of SICK-Dominant, Normal-Dominant, and Normal-Non-Dominant UT were significantly increased (*P*<0.05). The maximal MA of Normal-Dominant LT was significantly increased (*P*=0.029), and the SICK-Dominant and Normal-Dominant groups showed significantly higher maximal and mean MAs after exercise compared with the Normal-Non-Dominant group (*P*<0.05). The maximal MA of SICK-Dominant musculi serratus anterior was significantly lower than Normal-Dominant at pre-test (*P*=0.034), and the mean MA of SICK-Dominant musculi serratus anterior differed from Normal-Dominant and Normal-Non-Dominant (*P*<0.05) before testing, but only from Normal-Non-Dominant after testing (*P*=0.031).

**Conclusion:**

Scapular kinetic-chain exercise improved muscle activation in both overhead-pitching players with scapular dyskinesis and normal players.

## Introduction

The shoulder consists of 3 bones (the clavicle, scapula, and humerus) and 4 joints (the sternoclavicular, acromioclavicular, glenohumeral, and scapulothoracic joints). The glenohumeral joint is generally referred to as the shoulder joint. The shoulder joint has the widest range of motion (ROM) due to the small glenoid and relatively large humeral head (1), and about 7% of all sports injuries are related to the shoulder joint with a very high recurrence rate (2). Shoulder joint stability is maintained by static structures, including the scapula-humeral ligament, labrum, and joint capsule, and dynamic structures, including the rotator cuff and the long head tendon of the biceps brachii muscle (3). Shoulder joint movement is always involved in scapular exercise, and therefore, the movement and solidity of the scapula are very important for athletes as scapular exercise directly affects shoulder joint movement (4).

However, glenohumeral joint movement is lost if abnormal scapular movement makes the functional movement of the humerus impossible, resulting in an increased likelihood of injury to the scapula. This is very important to the functional role of the scapula in repetitive overhead motion, like pitching a baseball (5), and the accumulation of muscular or joint periphery tissue microdamage by excessive pitching can induce scapular dysfunction. Scapular exercise can lead to impingement syndrome, SLAP (superior labral tear from anterior to posterior) lesions, and scapular dyskinesis (6). Scapular dyskinesis is defined as movement out of the normal range of motion of the scapula, and abnormal findings involved in scapular dyskinesis are integrated into the SICK (S, scapular malposition; I, inferior border prominence; C, coracoid process pain; K, scapular dyskinesis) scapular syndrome (6). SICK is caused by fatigue from muscle overuse and is also referred to as “dead arm” (6).

Reinforcing the scapula muscle helps to increase the stability of the base of the humerus, which is essential in rehabilitating pitching players to enable optimum pitching motion (7–9). A scapulothoracic joint exercise program introduced (10) and a scapula stabilization exercise program were suggested to treat scapula dysfunction (6). The protocol in the current study is a “closed kinetic-chain” exercise that includes other segments in shoulder exercise and was formed on the basis that muscle insertion is fixed and that the muscle origin forms motion (11,12). The thoracolumbar fascia is shortened when the leg is extended and the gluteus maximus flexed during scapula stabilization exercise. The shortened thoracolumbar fascia is transferred to the opposite scapula (13), and this is why the lower limbs and trunk are used to deliver maximum strength and speed to the pitching arm during pitching motions (14).

Multilateral studies have been conducted to find effective scapula stabilization exercises regarding scapular dysfunction. However, significant effects of each exercise method were only found by integrating the upper and lower limbs, and studies directly applying to overhead-pitching players with scapular dysfunction are inadequate, suggesting the necessity of exercise rehabilitation programs for scapular stabilization to increase the actual effect of exercise rehabilitation.

Thus, the present study observed changes in muscle activation according to scapular kinetic-chain exercise participation in overhead-pitching players who participated in matches to provide basic data for scapular injury prevention and performance reinforcement.

## Methods

### Participants

A total sample size of 24 was calculated by measuring twice with G*Power software (version 3.1.7, Heinrich-Heine-University, Düsseldorf, Germany) set at a significance level of 0.05, power of 0.95, and an effect size of 0.40. Twenty-six subjects were recruited considering dropouts. Twenty-three subjects recruited through the Sports Science Institute of Korea National Sport University, Seoul, Korea in Sep 2014 were finally included in the study. There were 7 subjects with a SICK scapular rating of 10–14 and 16 of under 9 points as determined by an orthopedic specialist. A healthy scapula without any pain or asymmetrical symptoms scored 0 points, and a scapula with pain and abnormal structural position scored 20 points. Subjects with 10–14 points or 1–9 points were assigned to either a SICK or Normal group, respectively (6). These groups were further divided into SICK-Dominant, SICK-Non-Dominant, Normal-Dominant, and Normal-Non-Dominant to examine the dominant and non-dominant deviation of each group.

The subjects were baseball players with a career of more than 7 years with no orthopedic disease in the previous 6 months, no physical limitation in exercising, and who voluntarily gave informed consent. Subjects were not allowed drug treatment except for acute injuries during matches or training, and matches, training, and diet were controlled by dormitory life. All study participants provided informed consent, and the study design was approved by the Korea National Sport University. Subject characteristics are shown in Table 1.

**Table 1:** Subject characteristics (n=26)

***Variable***	***SICK (n=7)***	***Normal (n=19)***
Age (yr)	19.1±2.3	20.2±1.5
Career (yr)	9.7±1.5	10.37±2.0
Height (cm)	175.4±2.2	175.9±3.6
Weight (kg)	86.1±11.8	77.40±11.0
Body mass index (kg/m^2^)	28.0±4.0	25.0±3.6
Values are means±standard deviation		

SICK: S, scapular malposition; I, inferior border prominence; C, coracoid process pain; K, scapular dyskinesis

### Measurement of muscle activation

Electromyography (EMG) (Zerowire, Aurion Medical; Oderzo Traviso, Italy) and myoResearch XP (Noranxon; Scottsdale, AZ) were used for measuring muscle activity. Before attaching the electrode to the preselected muscle, body hair was removed to minimize noise and the skin was lightly wiped with alcohol to remove foreign substances or lotion. The skin surface was regarded as well-prepared if the skin resistance was 0.2–0.9 kΩ. If the skin resistance was found to be outside those values at any time, it was reevaluated to reduce EMG signal error. The distance between electrodes was 1.5 cm, and they were attached to the serratus anterior, upper trapezius, and lower trapezius to measure the electromyogram signals of dominant and non-dominant arms. Electrodes at the serratus anterior were in the middle of the muscle insertion between the left inferior margin of the scapula and the left part of the thorax. Electrodes at the upper trapezius were placed slightly interior between the posterior end of the acromial process and the 7^th^ cervical vertebral spinal process along the muscle belly of the trapezius. Electrodes at the lower trapezius were placed in an exterior superior diagonal direction along the line of intersection between the 8^th^ thoracic vertebral spinal process, the interior margin of the scapula, and the spina scapulae.

Maximal voluntary isometric contraction (MVIC) was applied to standardize EMG signals to compare subjects’ dominant and non-dominant EMG signals (15). EMG electrodes were attached to the serratus anterior and upper lower trapezius, and MVIC was conducted to measure % MVIC after attaching electrodes to each area. MVIC measurement was repeated 3 times by maintaining 5 sec of each motion, with 1 minute of resting time between each motion. This measurement was processed using the root mean square and the mean EMG signal of 3 sec (removing the first and last second of measurement) was used as 100% MVIC. The serratus anterior was measured at adduction (upper rotation of shoulder joint after 90° abduction and external rotation of shoulder joint), the upper trapezius at elevation of the scapula after cervical posteriolateral extension, and the lower trapezius at depression of the scapula after flexing the shoulder joint over the head to make it parallel to the lower trapezius fiber array. Three minutes of rest was given between measuring the dominant and non-dominant arms. Measurements were conducted after ensuring the subject understood the whole motion before the test. Mean and maximum values were calculated by measuring each motion 3 times.

### Rehabilitation exercise training

A scapula stabilization exercise program was conducted based on the program suggested (6). This consisted of 8 motions to reinforce muscles contributing to scapula stabilization, conducted 3 times a week for 8 wk with 10 min of stretching, 40 min of main exercise, and 10 min of cool-down. The exercise program is shown in detail in Table 2.

**Table 2:** SICK scapular rehabilitation exercise program

***Stage***	***Exercise***	***Time***	***Duration***
Warm-up	Shoulder stretching (pectoralis minor stretch, sleeper stretch, corner stretch)	-	10 min
Main exercise	One leg stance	10 repetitions, 5 sets	40 min
	Low-row	20 repetitions, 5 sets	
	Scapular clock	20 repetitions, 5 sets	
	Humeral head depression and rotation	10 sec, 10 repetitions, 5 sets	
	Wall washes	10 repetitions, 5 sets	
	Punches	20 repetitions, 5 sets	
	Push-up plus	20 repetitions, 5 sets	
	Scapular exercise (protraction, retraction, elevation and retraction, depression and retraction, internal rotation and elevation, external rotation and depression)	10 repetitions, 5 sets	
	Black burn	10 repetitions, 5 sets	
	Seated push-up	10 sec, 10 repetitions, 5 sets	
	Low-row with pulley	10 sec, 10 repetitions, 5 sets	
Cool-down	Shoulder stretching	-	10 min
	(pectoralis minor stretch, sleeper stretch, corner		
	stretch)		

SICK: S, scapular malposition; I, inferior border prominence; C, coracoid process pain; K, scapular dyskinesis

### Statistical analysis

All results were presented as means±standard deviations. Data analyses were performed using twoway analysis of variance with repeated measures. The analyses were performed using SPSS version 18.0 (IBM; Armonk, NY) and statistical significance was set at *P*<0.05.

## Results

### Change of maximum muscle activation in elevation motion between groups according to exercise participation

There was a significant difference in the maximum muscle activation of elevation motion of the upper trapezius according to exercise participation in Normal-Dominant subjects between pre-test (79.93±22.14 %MVIC) and post-test (91.39±28.00 %MVIC) measurements (*P*=0.049) (Table 3).

**Table 3:** Changes in muscle activation of peak of elevation between groups

	***Group***	***Pre***	***Post***	***F***		***Sig.***
Upper trapezius	SICK-Dominant	81.91±22.13	109.40±20.69	G	1.552	0.213
	SICK-Non-Dominant	84.77±19.15	104.91±25.28	T	10.528	0.002
	Normal-Dominant	79.93±22.14	91.39±28.00^#^	G×T	2.685	0.057
	Normal-Non-Dominant	81.08±25.34	78.00±27.21			
Lower trapezius	SICK-Dominant	39.84±27.25	52.24±23.20	G	1.018	0.393
	SICK-Non-Dominant	36.41±20.83	50.42±13.40	T	4.050	0.050
	Normal-Dominant	37.70±31.15	48.78±32.28	G×T	0.424	0.737
	Normal-Non-Dominant	33.03±18.60	35.01±20.90			
Serratus anterior	SICK-Dominant	8.94±5.29	13.48±7.76	G	1.585	0.205
	SICK-Non-Dominant	17.37±7.18	27.08±16.21	T	0.545	0.464
	Normal-Dominant	26.52±21.70	21.25±16.57	G×T	1.058	0.376
	Normal-Non-Dominant	25.51±23.74	25.99±19.56			

Values are mean±standard deviation

SICK: S, scapular malposition; I, inferior border prominence; C, coracoid process pain; K, scapular dyskinesis

G: group, T: time, G×T: group×time

# p<0.05 upper trapezius Normal-Dominant pre vs. post

### Change of mean muscle activation in elevation motion between groups according to exercise participation

There was a significant difference in mean muscle activation in the elevation motion of the upper trapezius according to exercise participation between pre-test (74.79±24.36 %MVIC) and post-test (104.04±21.54 %MVIC) measurements in the SICK-Dominant group (*P*=0.031), and in the lower trapezius between pre-test (30.57±17.80 %MVIC) and post-test (41.73±21.19 %MVIC) measurements in the SICK-Dominant group (*P*=0.005) (Table 4).

**Table 4:** Changes in muscle activation of mean of elevation between groups.

***Variable***	***Group***	***Pre***	***Post***	***F***		***Sig.***
Upper trapezius	SICK-Dominant	74.79±24.36	104.04±21.54^#^	G	1.998	0.127
	SICK-Non-Dominant	79.03±17.85	94.22±20.99	T	7.685	0.008
	Normal-Dominant	74.08±23.71	81.95±27.04			
	Normal-Non-Dominant	72.10±27.00	68.51±26.46	G×T	2.558	0.066
Lower trapezius	SICK-Dominant	30.57±17.80	41.73±21.19^##^	G	1.230	0.309
	SICK-Non-Dominant	29.73±17.09	45.36±14.36	T	5.372	0.025
	Normal-Dominant	31.20±29.54	41.72±29.34	G×T	0.463	0.709
	Normal-Non-Dominant	25.62±14.36	28.55±16.27			
Serratus anterior	SICK-Dominant	6.92±3.48	10.54±6.10	G	1.665	0.187
	SICK-Non-Dominant	15.02±6.72	20.69±9.90	T	0.102	0.751
	Normal-Dominant	21.49±17.30	17.10±14.17	G×T	0.805	0.497
	Normal-Non-Dominant	21.42±20.51	19.89±14.47			

Values are mean±standard deviation

SICK: S, scapular malposition; I, inferior border prominence; C, coracoid process pain; K, scapular dyskinesis G: group, T: time, G×T: group×time

# *P*<0.05 upper trapezius SICK-Dominant pre vs. post

## *P*<0.01 lower trapezius SICK-Dominant pre vs. post

### Change of maximum muscle activation in depression motion between groups according to exercise participation

The maximum muscle activation in the depression motion of the lower trapezius according to exercise participation was significantly higher in the SICK-Dominant (71.54±28.79 %MVIC) and Normal-Dominant groups (61.31±26.87 %MVIC) than in the Normal-Non-Dominant group at post-test measurement (*P*=0.010, *P*=0.025; respectively). The Normal-Dominant group showed a significant difference between pre-test (49.30±23.40 %MVIC) and post-test (61.31±26.87 %MVIC) measurements (*P*=0.029). The maximum muscle activation in the depression motion of the serratus anterior was significantly lower in the SICK-Dominant group (8.35±3.45 %MVIC) compared to the Normal-Dominant group (25.78±20.90 %MVIC) at pre-test measurement (*P*=0.034) (Table 5).

**Table 5:** Changes in muscle activation of peak of depression between groups.

***Variable***	***Group***	***Pre***	***Post***	***F***		***Sig.***
Upper trapezius	SICK-Dominant	26.07±18.68	49.60±13.74	G	3.115	0.035
	SICK-Non-Dominant	33.78±30.78	45.51±29.15	T	19.577	<0.001
	Normal-Dominant	16.33±9.82	40.53±26.41	G×T	0.441	0.725
	Normal-Non-Dominant	13.09±13.14	30.55±24.69			
Lower trapezius	SICK-Dominant	56.37±21.38	71.54±28.79^*^	G	0.918	0.439
	SICK-Non-Dominant	43.81±16.81	54.90±15.71	T	2.032	0.160
	Normal-Dominant	49.30±23.40	61.31±26.87^*#^	G×T	3.055	0.037
	Normal-Non-Dominant	56.08±33.96	43.52±20.39			
Serratus anterior	SICK-Dominant	8.35±3.45	12.82±7.48	G	3.184	0.032
	SICK-Non-Dominant	14.15±5.56	18.30±9.33	T	0.213	0.647
	Normal-Dominant	25.78±20.90^*^	23.69±15.92	G×T	0.218	0.883
	Normal-Non-Dominant	23.00±20.50	23.00±14.56			

Values are mean±standard deviation

SICK: S, scapular malposition; I, inferior border prominence; C, coracoid process pain; K, scapular dyskinesis G: group, T: time, G×T: group×time

* *P*<0.05 lower trapezius SICK-Dominant post vs. Normal-Non-Dominant post

Normal-Dominant post vs. Normal-Non-Dominant post serratus anterior SICK-Dominant pre vs. Normal-Dominant pre

# *P*<0.05 lower trapezius Normal-Dominant pre vs. post

### Change of mean muscle activation in depression motion between groups according to exercise participation

The mean muscle activation of depression motion of the lower trapezius after exercise was significantly increased in the SICK-Dominant (pre-test, 18.39±12.4 %MVIC; post-test, 40.18±14.91 %MVIC; *P*=0.017), Normal-Dominant (pre-test, 11.28±6.90 %MVIC; post-test, 29.61±22.22 %MVIC; *P*=0.005), and Normal-Non-Dominant groups (pre-test, 8.89±7.47 %MVIC; post-test, 24.39±22.79 %MVIC; *P*=0.010). The mean muscle activation of depression motion of the lower trapezius was significantly higher in the SICK-Dominant (60.04±22.43 %MVIC) and Normal-Dominant groups (52.55±24.06 %MVIC) compared to that in the Normal-Non-Dominant group (37.34±17.39 %MVIC) at post-test measurement (*P*=0.015 and *P*=0.026, respectively). The mean muscle activation of depression motion of the serratus anterior of the SICK-Dominant group (7.22±2.83 %MVIC) significantly differed from Normal-Dominant (20.23±13.61 %MVIC) and Normal-Non-Dominant groups (20.62±19.18 %MVIC) at pre-test measurement (*P*=0.049 and p=0.042, respectively), and it was significantly different between the SICK-Dominant (9.74±5.14 %MVIC) and Normal-Non-Dominant groups (19.97±13.47 %MVIC) at post-test measurement (*P*=0.031) (Table 6).

**Table 6:** Changes in muscle activation of mean of depression between groups

***Variable***	***Group***	***Pre***	***Post***	***F***		***Sig.***
Upper trapezius	SICK-Dominant	18.39±12.40	40.18±14.91^#^	G	2.687	0.057
	SICK-Non-Dominant	21.87±18.71	33.99±21.80	T	21.233	<0.001
	Normal-Dominant	11.28±6.90	29.61±22.22^##^	G×T	0.243	0.866
	Normal-Non-Dominant	8.89±7.47	24.39±22.79^#^			
Lower trapezius	SICK-Dominant	47.27±17.78	60.04±22.43	G	0.680	0.568
	SICK-Non-Dominant	36.61±15.43	47.54±12.80	T	2.413	0.127
	Normal-Dominant	41.05±19.97	52.55±24.06^#^	G×T	3.229	0.030
	Normal-Non-Dominant	48.16±32.22	37.34±17.39^*^			
Serratus anterior	SICK-Dominant	7.22±2.83	9.73±5.14	G	3.047	0.038
	SICK-Non-Dominant	13.12±5.38	16.93±9.38	T	0.051	0.822
	Normal-Dominant	20.23±13.61^*^	17.02±8.38	G×T	0.373	0.773
	Normal-Non-Dominant	20.62±19.18^*^	19.97±13.47^*^			

Values are mean±standard deviation

SICK: S, scapular malposition; I, inferior border prominence; C, coracoid process pain; K, scapular dyskinesis G: group, T: time, G×T: group×time

# *P*<0.05 upper trapezius SICK-Dominant pre vs; post Normal-Non-Dominant pre vs. post lower trapezius Normal-Dominant pre vs. post

## *P*<0.01 upper trapezius Normal-Dominant pre vs. post

* *P*<0.05 lower trapezius SICK-Dominant post vs. Normal-Non-Dominant post Normal-Dominant post vs. Normal-Non-Dominant post serratus anterior SICK-Dominant pre vs. Normal-Dominant pre SICK-Dominant pre vs. Normal-Non-Dominant pre SICK-Dominant post vs. Normal-Non-Dominant post

## Discussion

This study examined the effect of 8 wk of scapular kinetic-chain exercise involving lower limb motion on muscle activation in university baseball players diagnosed with scapular dyskinesis by a specialist. The scapula rotates upward about 60° when the shoulder joint moves to help smooth motion without collision between the humerus and acromion, and another role of the scapula is retraction and traction of the manubrium of the sternum. In overhead motion, or throwing motion, the scapula should be able to retract to promote a cocking position, and scapular traction should be available in the accelerating phase (16). The scapula also helps to elevate the acromion. Abduction of the shoulder joint at 180° should cooperate with the 60° rotation of the scapula and the 120° movement of the humerus, and impingement syndrome caused by collision of muscles and the humerus and acromion occurs if there is no scapular rotation during movement of the humerus due to incomplete elevation (17).

The upper and lower trapezii should work with the rhomboideus and the serratus anterior for scapular stabilization, with pairing between the lower trapezius and serratus anterior and the upper trapezius and rhomboideus for elevation (18). The mechanism behind abnormal muscle activation in scapular dyskinesis is divided into neuromuscular absence (lack of activation of contraction and coupling of force) or lack of activation. Weak muscle activation in the serratus anterior and lower trapezius can sometimes induce hyperactivity of the upper trapezius, and an absence of scapular retrodis-placement is linked to a loss of the cocking point, losing energy transfer from the body to the upper limb through the thorax. The 20% loss of energy transferred from the thorax to the arm leads to a 34% increase of rotation speed in the scapula to produce the same amount of energy (7, 19–22). The absence of elevation of the acromion due to elevation of the shoulder joint in the cocking and follow-through phases can result in a primary or secondary collision from instability due to fatigue and disturbance of the lower trapezius and serratus anterior, resulting in a relatively narrow coracoacromial arch. Thus, decreased activation of the serratus anterior in an overhead-pitching player with a shoulder lesion can explain a strength imbalance between the serratus anterior and trapezius (20), resulting in impingement syndrome due to abnormal muscle activation in the upper and lower trapezii and serratus anterior (19–20, 22–24).

Motions such as protraction/retraction and elevation/depression of the shoulder joint are important factors in minimizing fatigue and preventing injury in overhead motion. The serratus anterior, which attaches the scapula to the thoracic wall, is a very important muscle for protraction, and the middle and lower trapezii and rhomboideus play roles in anchoring the shoulder to the center of the body (25). The elevating muscle group consists of the upper trapezius, levator scapulae, and rhomboideus (26), and the depressing muscle group consists of the lower trapezius, latissimus dorsi, and teres minor (25).

In this study, there was a significant difference between pre- and post-testing in the maximum value of elevation motion in the Normal-Dominant group and the mean value of the SICK-Dominant group. In the case of depression motion, there was a significant difference between the Normal-Non-Dominant group and both the SICK-Dominant and Normal-Dominant groups in the maximum and mean values, and the Normal-Dominant group showed a significant difference between pre- and post-testing with exercise. The SICK-Dominant and Normal-Dominant groups showed a significant difference in the maximum values of retraction and depression motion before exercise, the mean value of depression motion of the SICK-Dominant group significantly differed from the Normal-Dominant and Normal-Non-Dominant groups before exercise, and there was a significant difference between the SICK-Dominant and Normal-Non-Dominant groups after exercise.

These results seem to be due to differences in depression motion by the deformation of force coupling between the lower and upper trapezii due to protrusion and abduction of the inferior angle, which are symptoms of SICK (6, 27), and due to higher muscle activation in retraction and elevation motion due to compensation by the upper trapezius in cooperation with the serratus anterior. However, SICK groups that showed overall scapular dyskinesis had a tendency to increase of muscle activation, but Normal groups showed a tendency to decrease of muscle activation.

In this study, the lower trapezius showed a difference in the mean value of elevation motion and the maximum and mean values of depression motion due to its role as an anchor in securing the stabilization of the scapula in elevation motion (9). In addition, there was proper muscle activation for fiber packing and motion direction owing to the activation of the lower trapezius in depression motion. Moreover, the serratus anterior showed significantly lower maximum values of retraction and depression motion, but overall tended to have slightly increased maximum and mean values in SICK groups.

We have the following limitations. First, the difference between these results seems to be because the exercise program effect was not maximized in 8 wk (a short training period) due to various external factors, including a lack of concentration when preparing for matches. Second, because the participants were only male players from Seoul, this study cannot be generalized to all Korean baseball players. Third, this study consisted of only a few baseball players (n=23). However, from this study, a more effective program could be developed for overhead-pitching players with functional problems of the scapula if future studies subdivide the rehabilitation phases of the exercise program.

## Conclusion

Scapular kinetic-chain exercise effectively improves muscle activation in overhead-pitching baseball players with scapular dyskinesis as well as normal players. As it is difficult to conclude that an upper limb rehabilitation exercise program with lower limb motion is more directly effective compared to upper limb-limited exercise, additional studies with an increased exercise period or subdivision of rehabilitation phases in exercise programs are necessary.

## Ethical considerations

Ethical issues (Including plagiarism, informed consent, misconduct, data fabrication and/or falsification, double publication and/or submission, redundancy, etc.) have been completely observed by the authors.
